# Interventions to reduce stress and prevent burnout in healthcare professionals supported by digital applications: a scoping review

**DOI:** 10.3389/fpubh.2023.1231266

**Published:** 2023-10-25

**Authors:** Daniela Adam, Julia Berschick, Julia K. Schiele, Martin Bogdanski, Marleen Schröter, Melanie Steinmetz, Anna K. Koch, Jalid Sehouli, Sylvia Reschke, Wiebke Stritter, Christian S. Kessler, Georg Seifert

**Affiliations:** ^1^Institute of Social Medicine, Epidemiology and Health Economics, Charité Universitätsmedizin Berlin, Corporate Member of Freie Universität Berlin, Humboldt-Universität zu Berlin, and Berlin Institute of Health, Berlin, Germany; ^2^Department of Pediatrics, Division of Oncology and Hematology, Charité Universitätsmedizin Berlin, Corporate Member of Freie Universität Berlin, Humboldt-Universität zu Berlin, and Berlin Institute of Health, Berlin, Germany; ^3^Department of Gynecology with Center of Surgical Oncology, Charité-Universitätsmedizin Berlin, Corporate Member of Freie Universität Berlin, Humboldt-Universität zu Berlin, and Berlin Institute of Health, Charité Medical University, Berlin, Germany; ^4^Department of Internal Medicine and Nature-Based Therapies, Immanuel Hospital Berlin, Berlin, Germany

**Keywords:** burnout, stress, healthcare professionals, prevention, digital applications

## Abstract

**Aim:**

Healthcare professionals are at increased risk of burnout, primarily due to workplace-related stressors. The COVID-19 pandemic has further increased this risk. Different interventions exist with varying degrees of effectiveness; little is reported on the content and implementation of such programs. This review fills this gap, with attention to recent programs using digital components.

**Methods:**

PubMed, Embase, PsycInfo, and Google Scholar were searched between January 24th and 28th, 2022, limited to the last 5 years (≥2017). Articles were included if they (1) focused on stress reduction or burnout prevention for nurses and medical doctors within workplace health promotion for nurses or medical doctors, (2) included a digital program component, (3) were conducted in high-income country contexts, and (4) were clinical studies published in English or German. Data was extracted using *a priori* designed spreadsheets. A group of at least 2 authors at each stage carried out the screening, selection, and data extraction.

**Results:**

The search strategy identified 153 articles, all except 7 were excluded. Two studies were conducted in the USA, two in Spain, one in the Netherlands, Poland, and Korea each. Four studies used a randomized study design, all but one had a control group. A wide range of outcome measures was used. The types of interventions included an adapted mindfulness-based stress reduction program combined with aspects of behavioral therapies, cognitive behavioral therapy, or acceptance and commitment therapy. The digital components used were apps (4 studies), a digital platform, blended learning, and a web-based intervention (1 study each). Six studies focused on individual interventions, one included organizational interventions.

**Conclusion:**

Despite an acute burnout crisis in the healthcare sector, only seven recent interventions were found that integrated digital components. Several problems emerged during the implementation of the interventions that made it clear that organizational support is urgently needed for successful implementation. Although interventions for stress reduction and burnout prevention should combine individual and organizational measures to be as successful as possible, this was only partially the case in one of the intervention programs. The results of this scoping review can be used to further develop or optimize stress and burnout prevention programs.

## 1. Introduction

Burnout is a work-related syndrome involving emotional exhaustion, depersonalization, and reduced personal accomplishment (1). In the updated International Classification of Diseases (ICD-11), burnout is listed in the section on problems related to employment or unemployment and is thus now classified as a syndrome. In the workplaces of healthcare professionals, stressors such as a high workload, dealing with suffering patients, and other workplace related conflicts are among the greatest risk factors for developing burnout (2–4). The COVID-19 pandemic has further intensified this psychological burden for healthcare professionals (5, 6). The 2020 Medscape National Physician Burnout and Suicide Report estimated a burnout rate of approximately 43% with women and middle-aged (40–54 years) physicians reporting more commonly symptoms of burnout (7). Estimates of prevalence among medical doctors in Germany vary between 4 and 20%, suggesting much higher values for burnout than in the general population (4.2%) (8). Physician burnout is a global crisis (9). In healthcare professionals burnout not only results in negative health consequences for the individual (10); it also leads to lower patient safety, poorer patient care, lower professionalism, more workplace injuries and higher absenteeism (10–18). The economic impact of burnout among healthcare workers is immense due to increased absenteeism and turnover (19–24), further exacerbating the already acute labor shortage (25, 26).

Current systematic reviews and meta-analyses on interventions for stress reduction and burnout prevention in healthcare professionals have shown that many different interventions are available with varying levels of effectiveness (27–30). Conclusions suggest that individual and organizational solutions are ideally combined to obtain greater improvements in well-being, that interventions should be easily accessible at work, and that digital technologies may be promising support tools in stress prevention (27–30). Comparatively little has been reported regarding detailed intervention characteristics, the digital applications used and how those are integrated into workplace environments. Digital technologies can be a valuable support not only in patient care but also in interventions directed directly to healthcare professionals (31). However, this is needed to further develop the quality of the interventions, especially regarding the integration of digital components.

The goal of this scoping review is to identify current stress reduction and burnout prevention interventions supported by digital components for nurses or medical doctors within workplace health promotion. Of interest were specific programs dedicated to burnout prevention – i.e. in the sense of primary prevention to avoid the development of an illness – as well as programs aimed at reducing stress. Although stress does not *per se* lead to burnout or other stress consequences, too much stress or not having enough resources to cope can lead to negative consequences (32). These interventions should take place in the context of the general workplace health promotion activities of the respective hospitals. Detailed information on program content, implementation processes, evaluation methods, and relevant endpoints will be provided for further development or optimization of prevention programs in this field. Due to the COVID-19 pandemic situation since early 2020 and the increasing demand for digital applications, the integration of digital applications into interventions is evaluated.

## 2. Methods

This scoping review adheres to the recommendations from the *Preferred Reporting Items for Systematic Review and Meta-Analysis extension for Scoping Reviews* (33). A protocol describing rationales and planned methods was submitted to osf.io for registration.^1^

### 2.1. Eligibility

Inclusion and exclusion criteria were determined based on the research question and discussed in the research team (Table 1). To be included, studies had to evaluate recent workplace health promotion programs using digital components (web-based, digital platforms and apps) for nurses or physicians that were designed to reduce stress or prevent burnout as part of workplace health promotion. Due to the special requirements of these professions, we focused our search on this clientele. Clinical trials from countries in the high-income country context were included assuming that these are provided with similar health care resources. Databases PubMed, Embase, and Psycinfo were searched. Articles published from 2017 onwards have been screened to include only the most recent results. To identify publications that may not have yet been indexed in any of the databases, the search was expanded for Google Scholar limited to the most recent years (≥ 2020).

**Table 1:** Inclusion & exclusion criteria.

	Inclusion	Exclusion
Study population	Nurses and medical doctors	Other healthcare professionals
Health area	Workplace health promotion	Other health areas
Content of interest	Programs developed to reduce stress or prevent burnout for nurses and medical doctors within workplace health promotion using digital support	Review of App functionality, usability survey results
Digital type	Web-based (accessible via world wide web) or digital platforms (accessible via company intranet), apps	Telehealth, Text/SMS-based health, video conferencing, health product-based, tracking devices, and virtual reality
Publication type	Clinical studies	Reports, study syntheses, theses, epidemiological studies, guidelines, handbooks, instructional manuals, user-based information, technical or specialist publications, commentaries, and product descriptions
Countries of interest	High-income country context according to The World Bank income groups (34)	≤ Middle income country contexts
Database and timeframe	PubMed, Embase, Psycinfo: ≥ 2017 Google Scholar: ≥ 2020	
Language	English or German	

### 2.2. Information sources and search strategy

A search strategy was developed by the research team that consisted of psychologists, physicians and other experienced researchers. The search consisted of associated terms and their keywords, according to the thesaurus of the underlying databases, for “occupational health management,” “stress” or “burnout” (indication), “health care professionals” and “digital component.” A detailed list of German and English search terms and Boolean operators is found in Table 2. PubMed, Embase, PsycInfo, and Google Scholar were searched between 24th and 28th January 2022. A grey literature search on stress prevention projects in hospitals was conducted in parallel but will be published in a separate article due to the limited scope of this review.

**Table 2:** Search strategy.

Occupational health management	Betriebliches Gesundheitsmanagement OR occupational health management OR occupational healthcare management OR corporate health management OR corporate healthcare management OR health care management [Emtree] OR Betriebliche Gesundheitsfoerderung OR workplace health promotion OR occupational health promotion OR occupational health [MESH Term] OR health promotion [MESH Term / Emtree] OR occupational health service [Emtree]
	AND
Indication	Stressreduktion OR stress reduction OR stress [APA Thesaurus of Psychological Index Terms] OR stress, psychological [MESH Term] OR occupational stress [MESH Term] OR physiological stress [MESH Term / Emtree] OR psychological stress [MESH Term / Emtree] OR mental stress [Emtree] OR emotional stress [Emtree] OR interpersonal stress [Emtree] OR job stress [Emtree] OR Stressprävention OR stress prevention OR Burnoutprävention OR burnout prevention
	AND
Health care professionals	Gesundheitsberufe OR health care professionals OR healthcare professionals OR Health Personnel [MESH Term] OR health care personnel [Emtree] OR Arzt OR medical doctor OR physician OR medical practitioner OR doctor OR physicians [MESH Term] OR doctor, physicians [Emtree] OR medical personnel [MESH Term / Emtree] OR practitioner [Emtree] OR private physician [Emtree] OR physician associate [Emtree] OR Krankenpflege OR nurse OR nursing [MESH Term] OR nurses [MESH Term] OR paramedical personnel [Emtree] OR paramedics [APA Thesaurus of Psychological Index Terms]
	AND
Digital component	Digital OR digital technology [MESH Term / Emtree] OR Digital Interventions [APA Thesaurus of Psychological Index Terms] OR Digital Health Resources [APA Thesaurus of Psychological Index Terms] OR app OR application OR Mobile Applications [MESH Term] OR mobile application [Emtree] OR mobile health application [Emtree]

### 2.3. Data collection and synthesis

Identified publications were imported into EndNote (version 20.2.1), duplicates were removed. Two authors (JB & DA) independently reviewed the abstracts and titles. Then, full-text articles were screened (JB & DA). Two electronic spreadsheets were developed *a priori* for data extraction. The first form contained data on general study characteristics. The second form contained data on intervention characteristics. JB & DA independently extracted the data into these two spreadsheets. Discrepancies were resolved through discussion between JB, DA, and the rest of the research team. The results of the data extraction, particularly related to the topics of intervention group content/exercise and implications for future interventions, were summarized and discussed by the reviewers (JB & DA) and other members of the research team.

## 3. Results

**Figure 1:**
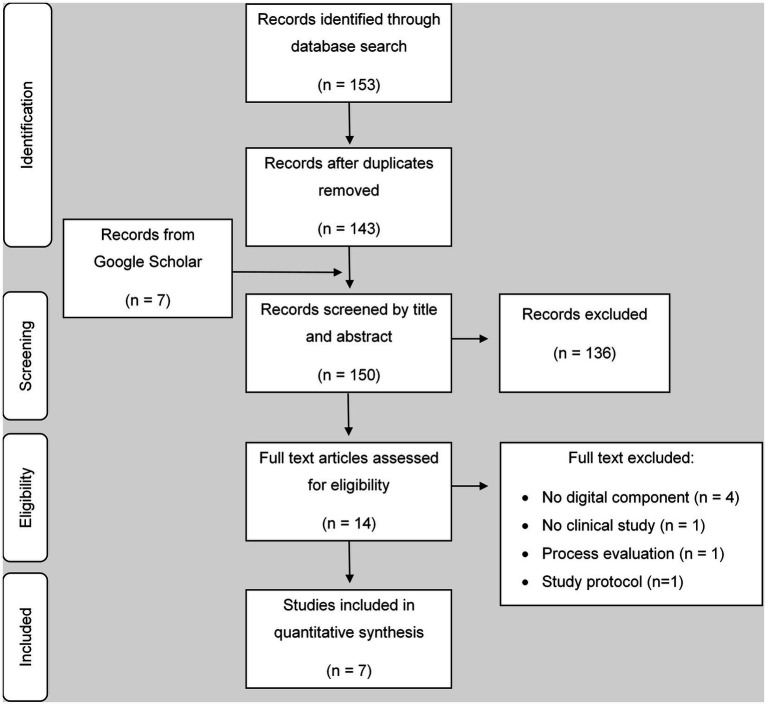
Flowchart.

The search strategy identified 153 publications in medical databases. After the removal of duplicates, 143 publications remained. The search in Google Scholar revealed seven additional publications. In total 150 publications were screened by title and abstract. 136 did not meet the inclusion criteria. Fourteen full text articles were screened for inclusion. Seven articles met all inclusion criteria and were used for further data extraction (Figure 1). All publications were original research articles (35–41). Reasons for exclusion of the other seven articles were: no digital component was involved (*n* = 4) and no primary research article (*n* = 2) or only a study protocol was published (*n* = 1). One article described a process evaluation of a study with no results included (42). The primary research article was searched and included into the scoping review (37).

### 3.1. Study characteristics

#### 3.1.1. Country

General study details are presented in Table 3. Two out of seven studies were conducted in the USA (35, 39) and two were carried out in Spain (36, 40). The others were conducted in the Netherlands, Poland, and Korea (37, 38).

**Table 3:** Study characteristics.

Author	Country	Year	Objective	Design	Sample size	Outcome measures	Points of measurement	Results
Coifman et al. (35)	USA	2021	Test the efficacy of a brief online ambulatory intervention aimed at supporting psychological health and well-being during the COVID-19 pandemic	Randomized assigned, pre-post comparison	*N* = 28 (assigned to low dose or high dose intervention)	Primary outcomes: PE (happiness, amusement, affection, contentment, relief) and NE (disgust, anger, sadness, fear, distress)	Before and after (1 week) each session participants rated intensity of specific NE and PE	Low dose and high dose intervention: sig. Decrease in NE by 44% and increase in PE by 13%; Higher dose condition: additional 9.4% increase in PE (pre- to post-session) + NE decrease by an additional 7.8%
Fiol-DeRoque et al. (36)	Spain	2021	Evaluate the effectiveness of a psychoeducational, mindfulness-based mHealth intervention to reduce mental health problems during the COVID-19 pandemic	Blinded, individually randomized, parallel-group, controlled trial	Intervention group *n* = 248; Control group *n* = 234	Primary outcomes: DASS-21; secondary outcome: DTS, MBI-HSS, ISI, GSE, Individual subscales of the DASS-21, Usability of PsyCovidApp	Baseline and at 2 weeks	Primary outcome: no sig. Differences between the groups at 2 weeks; subgroup analyses: sig. Improvements among HCW consuming psychotropic medications + receiving psychotherapy.
Havermans et al. (37)	Nether-lands	2018	Investigate the effectiveness of a digital platform-based implementation strategy regarding stress, determinants of work stress and the level of implementation among HCW	Cluster-controlled study design with 30 teams matched and assigned to either the experimental or control group	Intervention group *n* = 252 (15 teams); control group *n* = 221 (15 teams)	Primary outcome: stress: DASS-21; Secondary outcomes: psychosocial work factors (psychological demands, social support, autonomy) measured using sub-scales from the JCQ	Baseline, 6 month, 12 month	Stress: overall effect of the strategy statistically sig., control group showed higher stress scores than the intervention group over time. Separate analyses follow-up: statistically sig. Effect for stress at 6, but not at 12 months.
Hwang and Jo (38)	Korea	2019	Develop an app for mental health and assess its feasibility and effectiveness regarding stress management	Pretest-posttest design (mostly randomly assigned)	Intervention group *n* = 26; control group *n* = 30	PSS-10; KOSS; PHQ-9; GAD-7; Korean Emotional Labor scale; WHO-5; Self-efficacy; level of satisfaction with the app	Before and after the intervention	Stress, depression, anxiety, and emotional labor improved; positive index of well-being and self-efficacy level increased.
Mistretta et al. (39)	USA	2018	Assess whether an in-person mindfulness-based resilience training (MBRT) program or a smartphone delivered resiliency-based intervention improved stress, well-being, and burnout.	3-armed RCT	Intervention group *n* = 22; smartphone resilience intervention app *n* = 23; control group *n* = 15	Primary outcome: DASS-21, WHO-5; secondary outcome: MBI-HSS, SCS, compassion for others scale, daily affect, relationship quality, valued action, sleep monitoring	Baseline, 6 weeks (after the end of the intervention), 3 months	MBRT group: sig. Reductions in stress at 6 weeks and 3 months + sig. Improvement in emotional exhaustion subscale (MBI). MBRT and smartphone resilience group: sig. Increases in well-being at 6 weeks and 3 months.
Montero-Marin et al. (40)	Spain	2018	Investigate if blended mindfulness-based program for GPs, without support or guidance, would improve well-being and if the construct of awareness would be a mediating factor for improvement	Open uncontrolled trial	*N* = 290	Primary outcome: PANAS-positive; secondary outcomes: PANAS-negative, MAAS, CDRISC, BCSQ-12	Before and after the intervention	Participants completing “two or more weekly practices”: pre-post improvements for PANAS-positive + sig. Improvements in MAAS. Participants completing “one weekly practice”: no sig. Improvements in PANAS-positive. No sig. Improvements in PANAS-negative, CDRISC or BCSQ-12 in any group.
Smoktunowicz et al. (41)	Poland	2019	Test if a Med-Stress internet intervention reduces job stress, burnout, depression, and job-related secondary stress and increases work engagement through the enhancement of perceived social support and self-efficacy.	4-arm parallel RCT	*N* = 1,200 (300 participants per condition)	Primary outcomes: PSS-14, OLBI; secondary outcomes: PHQ-9, UWES-3, PCL-5	Pre-intervention, post-intervention (6 weeks experimental groups; 3 weeks control groups), follow-ups: 6 month, 12 month	High dropout (loss to posttest 82.5%), Job stress: No sig. Effect of condition assignment (groups) on job stress for any between-group comparison, but sig. Effect of time for all comparisons (stress decreased with time). Burnout: no main effect of assignment on job burnout for all comparisons, but main effect of time for all comparisons (burnout decreased with time).

BCSQ-12, burnout clinical subtype questionnaire; CDRISC, Connor-Davidson resilience scale; DASS-21, depression anxiety stress scale-21; DTS, Davidson trauma scale; GAD-7, generalized anxiety disorder; GP, general practitioner; GSE, general self-efficacy scale; HCW, health care workers; ISI, Insomnia severity index; JCQ, job content questionnaire; KOSS, Korean occupational stress scale; MAAS, mindful attention and awareness scale; MBI – HSS, Maslach burnout inventory – human services survey; MBRT, mindfulness-based resilience training; *n*, number of participants per group; *N*, total number of study participants; NE, negative emotions; OLBI, Oldenburg burnout inventory; PANAS, positive and negative affect schedule; PCL-5, posttraumatic stress disorder checklist 5; PE, positive emotions; PHQ-9, patient health questionnaire; PSS-10, perceived stress scale 10; PSS-14, perceived stress scale 14; RCT, randomized controlled trial; SCS, self-compassion scale; sig., significant; UWES-3, Utrecht work engagement scale; WHO-5, WHO-five well-being index.

#### 3.1.2. Study designs

All studies were published between 2018 and 2021. Four studies used a randomized study design (35, 36, 39, 41). All studies but one had a control group (40). Six out of seven studies included one or more control groups (35–39, 41). Two studies applied a waiting list design (37, 38). Four studies compared the intervention to active control groups (35, 36, 39, 41).

#### 3.1.3. Sample sizes

The sample size in the studies varied between *n* = 28 and *n* = 1,200 participants. Three studies had a rather small number of participants per group ≤30 (35, 38, 39). The four remaining studies included between *n* = 221 and *n* = 300 participants per group (36, 37, 40, 41).

#### 3.1.4. Outcome measures

A wide range of outcome measures were used. For the primary outcome, three studies used the Depression Anxiety Stress Scale-21 (36, 37, 39), two examined positive and negative emotions with different questionnaires (35, 40) and two focused on stress applying the Perceived Stress Scale (38, 41). For secondary outcomes, two studies used the Maslach Burnout Inventory – Human Services Survey (36, 39) and two made use of the Patient Health Questionnaire (38, 41). Results varied across the different studies and outcome measures.

#### 3.1.5. Results

Results of the Depression Anxiety Stress Scale-21 varied across the studies but were overall positively directed. Fiol-DeRoque et al. (35) found no significant differences between the control and intervention groups but subgroup analyses showed significant improvements among health care workers consuming psychotropic medications and receiving psychotherapy. Havermans et al. (36) analysed a stress subscale of the Depression Anxiety Stress Scale and found that the control group showed higher stress scores than the intervention group over time. Mistretta et al. (39) found significant reductions in stress for the Mindfulness-based resilience training. Further results of the primary and secondary outcomes of the studies are displayed in Table 3.

### 3.2. Intervention characteristics

Details on the interventions are presented in Table 4.

**Table 4:** Intervention characteristics.

Author	Intervention	Control	Duration intervention	Intervals intervention	Target population	Digital component	Implications for future interventions
Coifman et al. (35)	Expressive writing, adaptive emotion regulation activity, positive emotion-generation activities. An example of a prompt for positive emotions: “Think of a recent moment when you laughed and remember what was so funny”	Active control group: Equal to intervention only longer (“high dose” 4–6 min)	3–6 min	1 qd for 1 week	Medical and emergency personnel (hospital), personnel from police and fire departments	Smartphone app (intervention and control group)	Brief, daily, and ambulatory interventions are efficient.
Fiol-DeRoque et al. (36)	PsyCovidApp: Self-managed psychoeducational intervention, based on CBT and mindfulness: Written and audiovisual content targeting four areas: emotional skills, healthy lifestyle behavior, work stress and burnout, and social support (each with input to monitoring mental health status; education about psychological symptoms like anxiety; tips to manage pandemic-related stressors for example by relaxation and breathing techniques; healthy lifestyle and tips to promote it; organizational and individual strategies to promote resilience and reduce stress at work and burnout; and promotion of social support)	Active control group: General recommendations about mental health care (App)	Varied	Varied, app available for 2 weeks	HCW of COVID-19 patients’	Smartphone app (intervention and control group)	Use of passive comparator + a longer intervention period may be recommended; a digital intervention only may not produce significant improvements.
Havermans et al. (37)	Stress Prevention@Work: Multifaceted, integral implementation strategy: Digital platform including information, screening, planning tools + a search engine for interventions to prevent work stress (ranging from written guidelines to tailor-made prevention projects)	Waiting list	Varied	*Not stated*	Employees of a healthcare organization	Digital platform (intervention and control group)	Personnel shortage, turnover, and organizational restructuring hindered the use of the strategy.
Hwang and Jo (38)	*Not stated*	Waiting list	>10 min	More than twice per week, app available for 4 weeks	Nurses employed at hospitals	Smartphone app (intervention and control group)	Future research should include a wider sample, continuous counseling and health information provision via the app’s current functions, and the analysis of more factors.
Mistretta et al. (39)	MBRT (aspects of MBSR + ACT): MBRT involves learning mindfulness skills to effectively deal with unpleasant or unwanted thoughts or experiences, and learning resilience skills to promote positive growth and behavior in alignment with one’s intentions and values. It incorporates aspects of mindfulness-based stress reduction (MBSR) and Acceptance and Commitment Therapy (ACT), while differing from the two approaches in shorter meditation exercises and a deeper exploration of the neurobiology of stress and resilience. Live sessions topics (including education, practice, discussion): resilience, mindfulness, coping difficult physical sensations / emotions / unwanted thoughts, self-compassion; audios for self-training provided	Active control group: Smartphone Resilience Training: monitoring sleep, emotions + prompts (every 7–10 days) to select training topics: sleep, happiness + productivity, energy, focus, productivity. Control group with no additional intervention.	120 min	1 weekly for 6 weeks	Clinic employees	Smartphone app (control groups only)	Utility in both in-person delivered and smartphone-delivered mindfulness interventions + broader benefits regarding stress and work-related burnout for in-person MBRT interventions.
Montero-Marin et al. (40)	Blended, abbreviated web-based MBI: Face-to-face meeting: mindfulness theoretical module (usefulness for GPs, implementation in personal practice, incorporation into daily life), practical module (e.g., raisin exercise, mindfulness of breathing, body scan, 3-min practice, values-based practice adapted from ACT). Online training: audio + video practices (handling thoughts and emotions, walking meditation, mindful movements, kindly awareness meditation) + extended theoretical descriptions (texts + articles)	*No control group*	Face-to-face meeting: 4 h, online training: 45 min	All together: 10 h over a 1 month period, face-to-face meeting: once only, online training: 2 times per week over 4 weeks	General practitioners	Blended learning: face-to-face meeting combined with online training	Adherence in online / digital components are important for program success, blended formats are feasible.
Smoktunowicz et al. (41)	Med-Stress: Self-guided internet intervention, containing 2 main modules: SE and perceived SS. 3 CBT-framed exercises per module: consisting of psychoeducational animated clips + interactive tasks requiring both web-based and offline activities. SE exercises: mastery experience, vicarious experience, action planning. SS exercises: perceived support and cognitive distortions, social skills and peer support, action planning	Two active control groups: self-efficacy module only or perceived social support module only	1.5 h	Weekly over 6 weeks	Physicians, nurses, midwives, physical therapists, paramedics	Internet-based Intervention (intervention and control group)	Job burnout did not depend on assignment to the study condition. However, allocation to study condition did have an effect on job stress.

ACT, acceptance and commitment therapy; CBT, cognitive-behavioral therapy; HCW, health care workers; MBI, mindfulness-based intervention; MBRT, mindfulness-based resilience training; MBSR, mindfulness-based stress reduction; qd, daily; SE, self-efficacy; SS, social support.

#### 3.2.1. Intervention content

The interventions were multifaceted and varied across the studies. One study investigated an intervention consisting of expressive writing, adaptive emotion regulation activity, and positive emotion-generation activities (35). Another study investigated a self-managed psychoeducational intervention based on Cognitive Behavioral Therapy (CBT) and mindfulness (36). One study evaluated a multifaceted, integral implementation strategy consisting of a digital platform including information, screening, planning tools and a search engine for interventions to prevent work stress (37). A further study evaluated aspects of mindfulness-based resilience training (MBRT) (39). One study investigated a blended, abbreviated web-based mindfulness-based intervention (40) and another study a self-guided internet intervention containing CBT-framed exercises like (41). One study did not provide details on the intervention used (38).

#### 3.2.2. Duration and intervals of the intervention

Duration and intervals of the intervention exercises varied from 3 min to 4 h and from daily to weekly intervals or intervals chosen by the participants.

#### 3.2.3. Digital components

Four studies worked with digital components for the intervention and/or control group (35, 36, 38, 39). Two studies made use of a web-based platform in different ways: Havermans et al. (37) adopted it to give information on implantation strategies for work stress prevention programs on an organizational level. Smoktunowicz et al. (41) offered a self-guided intervention via the web-based platform. One study applied a blended learning format, which is a combination of face-to-face meetings and online trainings (web-based) (40).

## 4. Discussion

### 4.1. Summary of findings

This scoping review shows that the topic of stress reduction and burnout prevention for health professionals is being addressed in recent workplace health management projects that include digital components. It provides information about the individual content, implementation, and evaluation for seven current programs published within the last 5 years with particular attention to the use of digital components, which is of increasing relevance considering the COVID-19 pandemic (31). Content, form of the digital component and the scientific evaluation of the interventions differed fundamentally (35–41). Regarding implementation, it is concluded that brief interventions can be effective, but higher effects might be achieved by higher dose interventions (35, 37, 39) and the behavior of the leader and the characteristics of the team (37) play an important role in project success. Given the scarcity of findings, it is difficult to provide more practical recommendations.

Although interventions for stress reduction and burnout prevention should combine individual and organizational interventions to be as successful as possible (29), this is only partially the case in the studies identified here (37). Individual interventions such as here positive emotion-generation activities (35), self-managed psychoeducational activities (36), MBRT (39) mindfulness (40) or CBT-framed exercises (41) are an important component in the field of stress reduction and burnout prevention, they can hardly bring about sustainable changes on their own. For this, supplementary action at the organizational level is necessary. This is because the main cause of burnout lies in the working conditions of the respective work environment (4). In healthcare professionals, a lack of individual coping mechanisms can promote burnout, but they are rarely the sole cause (4). The fact that stress reduction and burnout prevention still mainly takes place at the individual level, despite better knowledge, raises questions. One reason for this might be that such research and measures are rarely carried out by the most authoritative organizations (43). Research actors usually have no possibility to intervene in existing structures or processes and therefore start where they can: at the individual level. An institutional anchoring of stress reduction and burnout prevention with the backing of management is essential in order to be able to implement organizational measures successfully and sustainably (43).

The results of the evaluated interventions clearly show the challenges faced by program developers: Personnel shortage, turnover, and organizational restructuring hindered the use of the interventions (37) and may be reasons for high drop-out rates (40). Also fear regarding stigma related to mental health issues at work inhibits both treatment and disclosure in physicians (44). The best intervention is of no use if it cannot be implemented in everyday life due to external factors. This must be considered when developing interventions and must not be neglected – an intervention that does not have the support of the executives and management will have a hard time succeeding in the long term. Support from management is not only directly necessary for the success of prevention programs but is also directly related to employee satisfaction. Studies have shown that dissatisfaction with workplace physical health protections was significantly associated with higher levels of emotional exhaustion (45). These problems are also reflected in the high drop-out rates; in one study it was as high as 82.5% (41). Statistical dropout analyses showed that dropouts were related to intervention assignment and occupational and demographic characteristics. Intervention modules that required less time were more likely to be completed. Older workers who had been on the job longer were also more likely to complete the study. These also had higher initial expectancy and higher perceived credibility of the intervention. Interestingly, those who had participated in the intervention had lower baseline scores for work stress, job burnout, depression and work-related post-traumatic stress. This suggests that those who started the intervention already very stressed and with high levels of burnout did not find the time to complete it. Of course, it is those who would benefit the most. Here it is up to the employer, despite acute staff shortages, to create structures that enable participation – at best also in the interventions that take more time. Although high dropouts are not uncommon in non-supervised Internet interventions, this is a particularly high value. The main reasons given for this were initial enthusiasm and curiosity that diminished over time. This is a problem that generally confronts health interventions: How do we ensure that interventions have a lasting effect? Interventions for effective stress reduction and burnout prevention must not be developed detached from the actual context in which they are to be applied later (46). A detailed and comprehensive needs assessment *a priori* with a targeted view also on the environmental factors of the respective work context is indispensable to be able to develop effective interventions. In the best case, these hindering environmental factors are then immediately integrated into the intervention. These findings complement the calls for a combination of behavioral and situational prevention: for effective stress reduction and burnout prevention, one is not possible without the other (46, 47). The health care system must meet these challenges in a timely manner. The consequences of burnout are severe, not only for healthcare providers but also for patients, as preventable medical errors become increasingly inevitable (10–13, 15, 16). A recent meta-analysis makes this point forcefully using data from 239.246 physicians (11): physician burnout undermines safe health care. Against the background of staff shortages in these important professions, this is once again a matter of urgency. Measures that are effective in practice are necessary and must no longer be regarded as nice-to-have. Without effective stress reduction and burnout prevention firmly anchored in occupational health management, our healthcare system is heading for disaster. The last few years, with the COVID-19 pandemic, have shown how quickly the demands on these professions can intensify again. Effective coping strategies are needed here, including effective stress reduction and burnout prevention programs.

While it is difficult to provide more practical recommendations given the scarcity of findings, the present scoping review illustrates that further research in this area is critically needed; especially regarding micro-interventions, wider samples, longer intervention periods including a passive comparator, interventions on organizational level and blended formats. For comprehensibility of the implementation of programs, they should be described as specific as possible in terms of methodical, didactical and content structure. Also, long-term acceptance (1–2 years perspective) is of importance.

### 4.2. Limitations

A limitation of the present review is that the quality of the studies included was not assessed; however, this is not required or advised for scoping reviews (33). Also, only seven studies were evaluated in total and thus only five countries reflected. Furthermore, we included only studies from high-income countries in German or English language and only recent publications on clinical studies since the year 2017. However, the overall project included a search for grey literature. It focused on stress prevention projects in hospitals which have not been evaluated in a clinical trial and contains results of semi-structured interviews with project members which gain more insight into this “unpublished” area. The density of information exceeded the scope of this review. Results have been accepted for publication recently (48).

### 4.3. Conclusion

There is a high demand for comprehensive support of healthcare professionals in terms of long-time mental health (3, 49). This review provides detailed information on the content of current international stress and burnout prevention programs with digital components for health professionals. Both potential barriers and potentially enabling factors related to program content and implementation are identified. The results of this scoping review can be used to inform prevention program development and research in this area. The fact that only seven recent interventions were identified in this scoping review, despite the acute health care burnout crisis, highlights the urgency of developing more programs that are effective, especially in real work settings.

## Author contributions

DA, JuS, JB, WS, SR, CK, and GS: conceptualization. DA, JuS, and JB: formal analysis. GS and CK: funding acquisition. DA, JuS, SR, and JB: investigation. DA, WS, and JuS: methodology. DA, AK, MeS, WS, GS, and CK: project administration. DA, AK, GS, and CK: supervision. DA and JuS: writing – original draft. JB, MB, MeS, AK, MaS, JaS, SR, WS, CK, and GS: writing – review and editing. No one was financially compensated for their contribution. All authors contributed to the article and approved the submitted version.

## Footnotes

^1^ https://osf.io/gsvcb
